# Low Pre-Transplant Caveolin-1 Serum Concentrations Are Associated with Acute Cellular Tubulointerstitial Rejection in Kidney Transplantation

**DOI:** 10.3390/molecules26092648

**Published:** 2021-04-30

**Authors:** Florian Emmerich, Stefan Zschiedrich, Christine Reichenbach-Braun, Caner Süsal, Susana Minguet, Marie-Christin Pauly, Maximilian Seidl

**Affiliations:** 1 Institute for Transfusion Medicine and Gene Therapy, Medical Center, Faculty of Medicine, University of Freiburg, 79106 Freiburg, Germany; christine.reichenbach-braun@uniklinik-freiburg.de (C.R.-B.); marie-christin.pauly@uniklinik-freiburg.de (M.-C.P.); 2 Nephrology, Department of Internal Medicine, Bürgerspital Solothurn, 4500 Solothurn, Switzerland; stefan.zschiedrich@spital.so.ch; 3 Department of Medicine, Renal Division, Medical Center, Faculty of Medicine, University of Freiburg, 79106 Freiburg, Germany; 4 Institute of Immunology, Heidelberg University Hospital, 69120 Heidelberg, Germany; caner.suesal@med.uni-heidelberg.de; 5 Signaling Research Centres BIOSS and CIBSS, Faculty of Biology, University of Freiburg, 79104 Freiburg, Germany; susana.minguet@biologie.uni-freiburg.de; 6 Centre for Chronic Immunodeficiency (CCI), Medical Center, University of Freiburg, 79106 Freiburg, Germany; 7 Institute for Surgical Pathology, Medical Center, Faculty of Medicine, University of Freiburg, 79106 Freiburg, Germany; maximilian.seidl@med.uni-duesseldorf.de; 8 Institute of Pathology, Heinrich-Heine University and University Hospital of Düsseldorf, 40225 Düsseldorf, Germany

**Keywords:** Caveolin-1, kidney transplantation, graft rejection, ischemia and reperfusion injury (IRI)

## Abstract

Acute and chronic transplant rejections due to alloreactivity are essential contributors to graft loss. However, the strength of alloreactivity is biased by non-immunological factors such as ischemia reperfusion injury (IRI). Accordingly, protection from IRI could be favorable in terms of limiting graft rejection. Caveolin-1 (Cav-1) is part of the cell membrane and an important regulator of intracellular signaling. Cav-1 has been demonstrated to limit IRI and to promote the survival of a variety of cell types including renal cells under stress conditions. Accordingly, Cav-1 could also play a role in limiting anti-graft immune responses. Here, we evaluated a possible association between pre-transplant serum concentrations of Cav-1 and the occurrence of rejection during follow-up in a pilot study. Therefore, Cav-1-serum concentrations were analyzed in 91 patients at the time of kidney transplantation and compared to the incidence of acute and chronic rejection. Higher Cav-1 levels were associated with lower occurrence of acute cellular tubulointerstitial rejection episodes.

## 1. Introduction

The success of organ transplantation depends essentially on the control of acute and chronic rejection of the graft. Suchlike alloresponses are initiated by the presentation of disparate alloantigens by either the donor’s or the recipient’s antigen presenting cells to the host’s immune system. Both pathways trigger the proliferation of allospecific T-cells and B-cells producing donor-specific antibodies directed against the graft endothelium [1,2]. However, alloreaction is not a stereotypical process and its strength is influenced by several non-immunological factors.

In particular, ischemia and reperfusion injury (IRI) plays a detrimental role in modulating adaptive and innate alloresponses [3,4,5,6,7]. This process is closely related to the up-regulation of Toll-like-receptor (TLR) expression in epithelial and endothelial cells during IRI [8].

Caveolins are components of the plasma membrane invaginations called Caveolae, which are engaged in endothelial cell function and signaling [9]. Caveolin signaling has been demonstrated to protect several cell types from IRI [10,11]. Since IRI is closely related to cell death, this effect might be due to the antiapoptotic function of Caveolin-1 [12]. In particularly, for renal cells the survival-promoting function of Caveolin-1 (Cav-1) has been demonstrated in response to apoptotic stimuli [13,14].

In addition, Cav-1-containing extracellular vesicles modulate cellular signaling pathways and crosstalk between endothelial cells and distal cellular system [15]. Therefore, we speculated that the presence of Cav-1 in serum might be relevant in renal transplantation.

In order to address this question, we conducted a pilot study and analyzed Cav-1 serum concentrations in a cohort of 91 patients prior to kidney transplantation and examined its association with HLA-mismatches (A, B, DR), patient sex, age at the time when the serum was drawn and histology, including histological features of graft rejection according to the Banff-classification [16,17]. Elevated Cav-1-serum concentrations correlated significantly with reduced incidence of cellular tubulointerstitial rejection episodes. Our data suggest that pre-transplantation Cav-1 serum concentrations could be a useful indicator for the identification of transplantation patients at risk of acute cellular tubulointerstitial rejection.

## 2. Results

### 2.1. Patients

Serum samples were collected between 2003 and 2017. Kidney biopsies were collected between 2004 and 2018. The cohort consisted of 91 HLA-matched patients, males (*n* = 60) being the majority, females (*n* = 31) the minority. A total of 77 patients received a first renal transplant, whereas 13 underwent a second and one a third transplantation. Only biopsies from the same kidney transplant with matching serum draw were considered. The data were aggregated on patients’ level (one biopsy per patient) or on the biopsy level (all biopsies). If more than one biopsy was available from the patient, those with the highest acute rejection category was chosen for the patients’ aggregation. Data of age at Cav-1 serum draw and kidney biopsies, Cav-1 serum level, active interstitial and tubular inflammation, acute vascular rejection, acute glomerulitis, mesangial matrix expansion and chronic interstitial scarring were available from all 91 patients. Data of transplant glomerulopathy were available from 90 patients, transplant vasculopathy and chronic tubular atrophy from 89 patients and HLA mismatches from 81 patients (HLA-A, -B and -DR). The overview of the descriptive statistics is summarized in Table 1 for the patients’ clinical characteristics and in Table 2 for the histological parameters; patients’ aggregation first, biopsy aggregation second.

**Figure 1:**
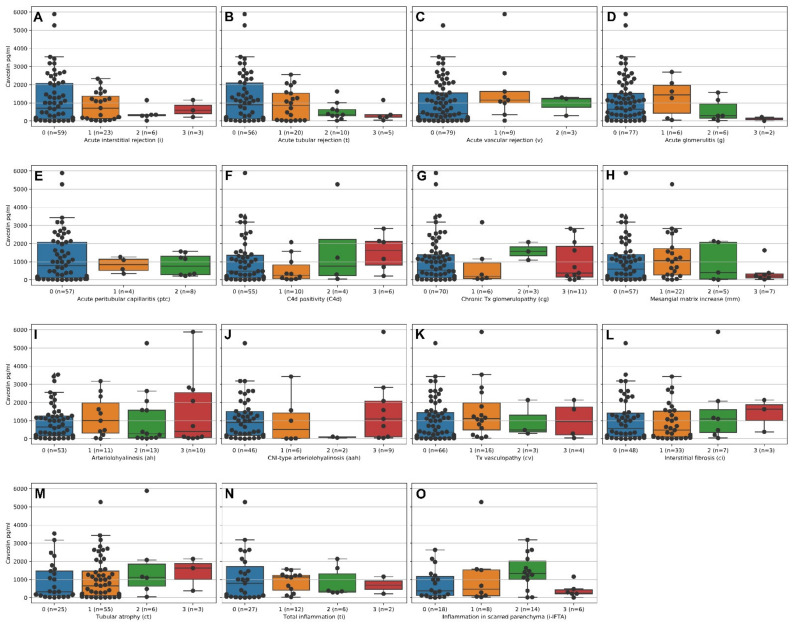
Bee-/Boxplots of Cav-1 serum levels in pg/mL vs. items according to the histological categories of the Banff classification. (**A**) Acute interstitial rejection, (**B**) acute tubular rejection, (**C**) vascular rejection, (**D**) acute glomerulitis, (**E**) acute peritubular capillaritis, (**F**) C4d positivity, (**G**) chronic transplant glomerulopathy, (**H**) mesangial matrix increase, (**I**) arteriolohyalinosis, (**J**) CNI-type arteriolohyalinosis, (**K**) transplant-vasculopathy, (**L**) interstitial fibrosis, (**M**) tubular atrophy, (**N**) total inflammation, (**O**) inflammation in scarred parenchyma.

Figure 1 gives an overview of the distribution of Cav-1 serum levels dependent on the different items of the Banff classification scheme [17]. The data is aggregated on patients’ level.

**Figure 2:**
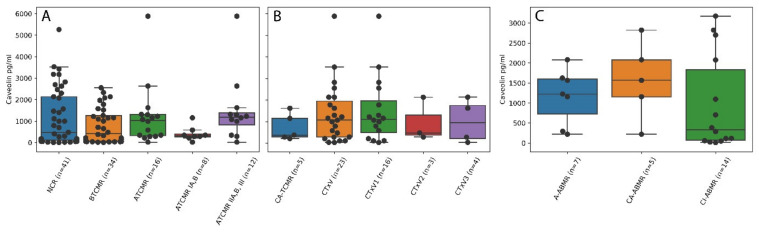
Overview of Banff categories/grades compared to Cav-1 serum levels. Boxes correspond to the 25% and 75% percentile, median line inside the box, whiskers display the rest of the distribution, outliers are outside the whiskers. (**A**) Acute cellular rejections: NCR = no cellular rejection; BTCMR = borderline (suspicious for) T-cell mediated rejection; ATCMR = acute T-cell mediated rejection; ATCMR IA,B = acute T-cell mediated rejection of grade IA,B (tubulointerstitial rejection); ATCMR IIA,B,III = acute T-cell mediated rejection of grade IIA,B,III (cellular vascular rejection). Values were available from all 91 patients. (**B**) Chronic damage: CA-TCMR = chronic and active T-cell mediated rejection (values were available from 46 patients); CTxV = chronic transplantation vasculopathy (cumulative and separated by intensity of fibrointimal narrowing from low/CTxV1 to high/CTxV3; values were available from 89 patients). (**C**) Antibody mediated rejection: A-ABMR = acute antibody mediated rejection; CA-ABMR = chronic active antibody mediated rejection; CI-ABMR = chronic inactive antibody mediated rejection. Values were available from 62 patients.

Figure 2 gives an overview of the distribution of acute and/or chronic cellular and humoral rejection categories and grades according to the latest Banff classification together with Cav-1 serum levels on the patients’ aggregation level. A table with the corresponding descriptive statistic is provided in the supplement (Supplemental Table S1). The acute T-cell-mediated tubulointerstitial rejection (ATCMR IA,B) score was classified as either no cellular rejection, borderline (suspicious for) TCMR or manifest ATCMR IA,B, which were defined through the Banff classification scheme. Accordingly, 41 patients did not show a cellular tubulointerstitial rejection, 34 patients displayed a borderline status, 8 patients showed a manifest ATCMR IA,B and 12 patients displayed an acute cellular vascular rejection/ATCMR IIA,B,III (Figure 2A). Some patients showed chronic rejection-associated damage, e.g., transplant vasculopathy was observed in 23 patients (Figure 2B), whereas fewer patients displayed acute and/or chronic humoral rejections (Figure 2C).

Data of medication were available for 89 patients in the induction phase, which comprised Cyclosporine A, Corticosteroids, Mycophenolate, Tacrolimus or Sirolimus: 38 patients received Cyclosporine, 83 Corticosteroids, 82 Mycophenolate, 43 Tacrolimus, 3 Sirolimus and 1 Azathioprine. 75 patients received a triple combination of Corticosteroids, Mycophenolate and Tacrolimus or Cyclosporine A or Azathioprine (1 patient). A total of 12 patients received a doublette consisting of Corticosteroids and/or Mycophenolate and/or Tacrolimus. One patient received only Tacrolimus, and one did not receive an induction therapy.

In the maintenance phase, medication data were available for 86 patients. Twenty-seven received Cyclosporine A, 74 Corticosteroids, 77 Mycophenolate, 50 Tacrolimus, 6 Sirolimus, 1 Leflunomide and 1 Azathioprine (which was not the same patient as in the induction phase). In total, 67 patients received a triple therapy combining the same medication as mentioned before. Thirteen patients received a doublette consisting of either Cyclosporine A, Corticosteroids, Mycophenolate, Tacrolimus, Sirolimus or Leflunomide. The patient with Leflunomide received Cyclosporine A, the patient with Azathioprine received Corticosteroids and Tacrolimus. One patient received only Corticosteroids, one did not receive a maintenance therapy (which was not the same patient as in the induction phase).

Eight patients underwent a change of the maintenance therapy, using the aforementioned immunosuppressive drugs. Azathioprine and Leflunomide were not used in this subgroup. One patient received a quadruple therapy, six patients received a triple therapy and one patient received a doublette.

### 2.2. Cav-1 Serum Concentration in Patients Prior to Kidney Transplantation and Its Correlation with Patients’ Medication

Serum concentration varied in the range of 3.6 to 5882.8 pg/mL which is in the range of findings in a previous report on healthy individuals [18]. The mean concentration and median were 1029.2 pg/mL and 586.7 pg/mL, respectively. Cav-1 serum levels were categorized in a four-tiered Cav-1 classifier (CC0-CC4) to harmonize the scale to the Banff classification scheme (see also Materials and Methods). According to the CC, the distribution was as follows: n_CC0_ = 67, n_CC1_ = 18, n_CC2_ = 4, n_CC3_ = 2.

As one patient in the CC1 group (induction phase) and one patient in the CC2/3 group (maintenance phase) were treated with Azathioprine, significant positive correlations exist between CC1, CC2/3 and Azathioprine in the induction and in the maintenance phase (r = 0.212 for CC1 and r = 0.396 for CC2/3). No further significant correlations could be observed between the immunosuppressive medication and CC0, CC1 and CC2/3.

### 2.3. Higher Caveolin Serum Levels Are Associated with Lower Intensities of Acute Cellular Tubulointerstitial Rejections (ATCMR IA,B) but Not Acute Vascular Rejection, Acute Humoral Rejection and Chronic Damage

**Figure 3:**
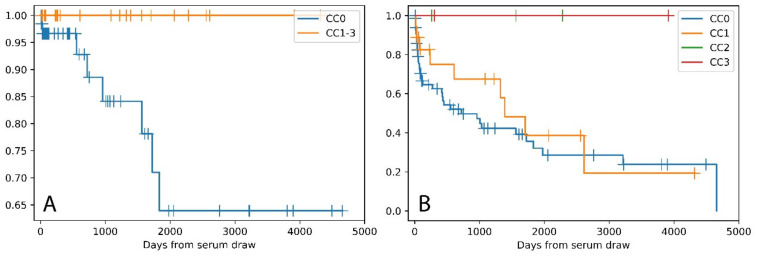
Kaplan-Meier curves of time from serum draw to ATCMR IA,B in the different Cav-1 classifier (CC) groups. (**A**) The eight events of ATCMR IA,B only occurred in the lowest CC group (CC0), *p* < 0.005 (logrank test). (**B**) Event was defined as borderline or ATCMR IA,B. The 42 events only occurred in the lower CC groups (CC0,1), *p* < 0.005 (multivariate logrank test).

Histological items classified according to the Banff classification scheme [17] were correlated with the CC, using the Spearman rho correlation coefficient. For analyses, data were aggregated (I) on patients’ (n_max_ = 91) and (II) on biopsy levels (n_max_ = 112). (I) Higher CCs are associated with lower ATCMR IA,B scores (ATCMR IA,B vs. CC: r = -0.213, *p* = 0.043; *n* = 91) but not with higher or lower ATCMR IIA,B,III scores (ATCMR IIA,B,III vs. CC: r = −0.004, *p* = 0.97). The CC positively correlates with the Tx age, measured in days from the serum draw to the biopsy (CC vs. days r = 0.213, *p* = 0.043, *n* = 91), indicating that higher CC is associated with higher Tx age. Therefore, a Kaplan-Meier analysis was conducted to display the time from serum draw to borderline or manifest ATCMR IA,B in the different CC groups (Figure 3), which significantly confirms the findings.

(II) Considering all biopsies, higher CCs are associated with lower ATCMR IA,B scores (ATCMR IA,B vs. CC: r = −0.244, *p* = 0.009; *n* = 112), lower acute interstitial rejection scores (i vs. CC: r = −0.19, *p* = 0.045; *n* = 112), lower acute tubular rejection scores (t vs. CC: r = −0.189, *p* = 0.046; *n* = 112), but not with acute vascular rejection scores (ATCMR IIA,B,III vs. CC: r = −0.066, *p* = 0.49). In contrast to these findings, analysis based on the Banff classification vs. the CC did not reveal any significant correlations for acute humoral rejections or parameters of chronic damage such as interstitial fibrosis or tubular atrophy, neither in the patients’ aggregation, nor in the correlation analysis comprising all biopsies. In the patients’ aggregation, a trend for a positive correlation between higher CCs and higher arteriolohyalinosis scores could be observed (ah vs. CC: r = 0.209, *p* = 0.052; *n* = 87).

A detailed list of the respective correlation coefficients together with *p*-values and numbers is given in the Supplemental Table S2, which also contains ratios of parameters over time. Significant positive correlations exist between acute interstitial, acute tubular and acute vascular rejections, respectively, either on the patients’ aggregation level or comprising all biopsies. Peritubular capillaritis significantly correlates both with parameters of acute cellular rejection and acute humoral rejection. Parameters of acute tubulointerstitial rejection do not significantly correlate with chronic tubulointerstitial atrophy/scarring. Acute glomerulitis significantly correlates with chronic lesions as transplant glomerulopathy and mesangiofibrosis (Supplemental Table S2).

### 2.4. Pre-Transplant Cav-1 Serum Levels Are Superior Indicators to Pre-Transplant HLA Mismatches for the Prediction of Acute Cellular Tubulointerstitial Rejections in the Posttransplant Setting

Data of HLA mismatches were available from 86 patients. None of the HLA mismatches (HLA-A, -B, -DR) significantly correlated with acute cellular rejections, which was also true for the sum of HLA mismatches (Supplemental Table S2). HLA-B mismatch negatively correlates with chronic transplant vasculopathy in the patient and biopsy-based aggregation (HLA-B mismatch vs. cv: r = −0.26, *p* = 0.017; *n* = 84 and r = −0.213, *p* = 0.03; *n* = 104). Positive correlations exist between sum of HLA-mismatches and age at serum draw and age at biopsy (Supplemental Table S2). To exclude biases due to differing scale levels or non-monotonic relations, the data including HLA-mismatch parameters were examined on patients’ aggregation level in an entropy reduction-based decision tree analysis as multivariable analysis to predict the classifiers “no ATCMR IA,B”, “borderline (suspicious for) ATCMR” or “ATCMR IA,B” (max. depth = 4, min. samples per leaf = 5, *n* = 79) including the following items: ‘HLA-mismatch A’, ‘HLA-mismatch B’, ‘HLA-mismatch DR’, ‘Sum mismatches’, ‘Cav-1 (pg/mL)’, ‘Sex (m = 1, f = 2)’, ‘Number of transplantations’, ‘Patient age at serum draw’, ‘Patient age at biopsy’, ‘Days between serum draw and biopsy’, ‘v’, ‘g’, ‘cg’, ‘mm’, ‘cv’, ‘ci’ and ‘ct’. The only HLA mismatch playing a role in our cohort was sum of HLA-mismatches as a level 3 predictor to separate no ATCMR IA,B and borderline ATCMR. Cav-1 serum level ≤ 2592.07 pg/mL was a level 1 predictor to separate no acute vascular rejection and no ATCMR IA, B (Supplemental Figure S1). To summarize, lower Cav-1 serum levels are superior indicators of ATCMR IA, B vs. HLA-mismatches, keeping in mind that the kidney transplantations in our cohort were performed under conditions of minimal HLA-mismatch between graft and host.

## 3. Discussion

In this work, we show that high serum concentrations of Cav-1 at the time of transplantation are associated with a reduced occurrence of acute cellular tubulointerstitial rejection (ATCMR IA,B) of renal grafts. Furthermore, we found a negative correlation between ATCMR IA,B and patients’ age at serum draw and biopsy, indicative of ATCMR IA,B being more frequent in “younger” patients of our cohort (Supplemental Table S2).

IRI has been shown to modulate both innate and adoptive immune responses and to promote long term graft damage [3,4,5,6,7]. One mechanism of action is the up-regulation of allogenic cell surface markers including MHC I and II, which may promote T-cell responses [19,20]. IRI also promotes the expression of toll-like receptors (TLRs) on epithelial and endothelial graft cells, which are in turn activated by endogenous ligands [8]. TLR recruitment has been demonstrated to be a major determinant of allograft damage and abrogation of TLR pathways induces rejection tolerance [21].

The resulting graft damage can be amplified by unmasking cryptic autoantigens released as vesicle-packed apoptotic particles, which are subsequently presented and trigger autoantibody production [22]. Indeed, a number of antibodies besides anti-HLA have been described to contribute to the complexity of allorecognition. The deleterious effects of non-HLA antibodies have been confirmed in patients with rejection and might explain the poor outcome even in the absence of HLA-donor specific antibodies (DSA) [23]. Accordingly, apoptosis protection of the graft could be factorable in terms of limiting allo- and autoimmune responses. Indeed, therapies enabling protection from IRI have been promising in attenuating anti-graft immune responses, thereby reducing the incidence of transplant complications [24].

Cav-1 has been demonstrated to protect renal cells from undergoing apoptosis under stress conditions [13,14]. One mechanism by which Cav-1 might exert its graft protective function is its negative effect on TGF-β-mediated signaling [25]. Particularly in renal cells, TGF-β amplifies apoptosis under stress conditions [26]. Of note, TGF-β has also been shown to promote fibrosis, a common complication after kidney transplantation [27].

In addition, the observation of attenuated T-cell mediated rejection might provoke the question of a potential immunosuppressive role of Cav-1 in T-cell activation. However, to the knowledge of the authors, this has not been addressed in the literature to the date of this submission.

Our findings raise the question as to how a host-derived protein can impact the survival of the transplanted tissue. One hint to answer this question might be the recent finding of intercellular trafficking of Cav-1 between distant cellular types such as adipocytes and endothelial cells [15]. This idea provides an attractive model as to how endogenous Cav-1 could be incorporated into the graft endothelium and subsequently execute beneficial functional effects to protect the graft.

Cav-1 has already been evaluated as a therapeutic target in several disease models [28,29,30,31,32]. In the context of the present work, it should be noted that a recombinant protein containing the caveolin scaffolding domain (CSD) protected renal endothelial cells from damage induced by angiotensin II (AT-II) [33]. This is of particular interest for patients with HLA-DSA negative humoral rejections, since AT-II type 1–receptor activating antibodies are strong inducers of allograft rejection and vasculopathy in patients without detectable HLA-DSA [34].

Our data propose Cav-1 serum concentrations as a potential tool for the identification of patients at increased risk of acute graft rejection. However, this study does not include a detailed analysis of the underlying kidney disease impacting transplant outcome. Furthermore, a higher temporal resolution and standardized manor of serum draws would lead to a better understanding of the kinetics of factors mediating transplant damage, as kidney biopsies might not be taken under these conditions due to ethical restrictions. It should be stressed as well, that a higher temporal resolution could better unveil non-monotonic relations, which could explain the discrepancy between factors of humoral rejection in the Spearman correlation (not significant) vs. the decision tree analysis (relevant to predict acute cellular rejection and stable over 10,000 runs).

Based on our pilot study, one could hypothesize that prospective Cav-1 measures could be useful to identify patients at risk for acute cellular tubulointerstitial rejection e.g., with only poorly matched organs. Limiting factors concern the patient numbers of our cohort with missing histological parameters due to historical reasons and lacking structured clinical data about the immunosuppressive regimen.

## 4. Materials and Methods

### 4.1. Patients’ Characteristics

The patients included in this study take part in the Collaborative Transplant Study (CTS) conducted by the Institute of Immunology, Heidelberg University, Germany, which includes written informed consent, ethical approval and allows subsequent analyses. Kidney transplantations were performed and serum samples were taken between 2003 and 2017 at the Medical Center Freiburg, Germany.

HLA typing data were taken from the ENIS database (Eurotransplant). According to Eurotransplant rules, HLA-antigen mismatches for HLA-A and -B were calculated based upon broad antigens and HLA-DR on split HLA antigens with the exception of DR17/DR18, which were regarded as broad DR3 antigen.

### 4.2. Caveolin Serum Analyses

Sera from patients of the study cohort with matching FFPE tissue samples in the pathological department (*n* = 91) (Institute for Surgical Pathology, University Medical Center Freiburg) were analyzed for Cav-1 levels by ELISA. ELISA was performed using the Human Cav-1 ELISA Kit (DEIA-XYA351V2) (Creative Diagnostics, New York, NY, USA). To increase the sensitivity, the assay was combined with the ELAST ELISA Amplification System (Perkin-Elmer; Waltham, MA, USA) following the manufacturer’s recommendations.

### 4.3. Histological Analyses

Periodic acid Schiff (PAS)-stained sections of 1.5 μm thickness were analyzed microscopically (Leica DM 2500; Leica, Wetzlar, Germany) and scored according to the Banff meeting report from 2015 where no previous classification was available from the reports (Loupy, Haas et al. 2017). All histological sections were derived from indication biopsies.

### 4.4. Statistical Analysis

Scientific data were collected in Microsoft Excel (Office 365 package). As the data were not normally distributed, statistical testing was performed by Spearman test for correlations coefficients, using a Python based solution (Python 3.7, Pandas, Numpy and SciPy package; script can be provided upon request by the authors). With respect to possible analytic conflicts due to different scales of the correlated categories (four possible stages in the Banff classification vs. theoretically unlimited positive stages in the values of Cav-1 serum-levels), a Cav-1 classifier (CC) was defined, reducing the number of different stages to four, as defined by the range of the Cav-1 serum-levels (min 3.6 pg/mL, max 5882.8 pg/mL): <1500 pg/mL = 0, 1500–2999 pg/mL = 1, 3000–4499 pg/mL = 2, >4499 pg/mL = 3. The number of days between serum draw/timepoint of transplantation and ATCMR IA,B was used to perform the Kaplan-Meier analysis (Python 3.7, Pandas, Matplotlib and Lifelines package). For multivariable analysis, an entropy-based decision tree classification was performed to look for non-monotonic relationships and/or relationships between variables of different scales (Python 3.7, Pandas, Numpy and SKlearn package). Parameters were included to predict the ATCMR IA,B as either “no cellular tubulointerstitial rejection”, “borderline (suspicious for) TCMR” or “acute TCMR grade IA,B”. Therefore, the values of active interstitial (i) and tubular (t) inflammation according to the Banff classification (Loupy, Haas et al. 2017) were summarized as square roots, which allows a clear numerical separation of the borderline categories.

## Figures and Tables

**Table 1:** Descriptive statistics of patients’ clinical values.

Clinical Data	*n*	Mean	std	Min	25%	50%	75%	Max
Age at Serum Draw	91	47.90	14.39	7	39	50	59.5	72
Age at Biopsy 1	91	50.10	14.34	7	43	51	61	72
Age at Biopsy 2	18	46.06	16.52	12	37.25	49	55	75
Age at Biopsy 3	2	40.00	35.36	15	27.50	40	52.5	65
Age at Biopsy 4	1	69.00		69	69	69	69	69
Sum HLA-Mismatch	86	2.71	1.63	0	2	3	4	6
HLA-Mismatch A	86	0.79	0.74	0	0	1	1	2
HLA-Mismatch B	86	0.95	0.65	0	1	1	1	2
HLA-Mismatch DR	86	0.97	0.69	0	0.25	1	1	2
Caveolin Classifier (CC)	91	0.35	0.67	0	0	0	1	3
Cav-1(pg/mL)	91	1029.23	1180.46	3.57	117.39	586.65	1543.82	5882.83
Antibodies developed (1 = yes, 0 = no)	86	0.17	0.38	0	0	0	0	1
Sex (m = 1, f = 2)	91	1.34	0.48	1	1	1	2	2

**Table 2:** Descriptive statistics of histological parameters.

Patient Aggregation	*n*	Mean	std	Min	25%	50%	75%	Max
One biopsy per patient	91	1.09	0.38	1	1	1	1	4
i	91	0.48	0.77	0	0	0	1	3
t	91	0.60	0.89	0	0	0	1	3
v	91	0.16	0.45	0	0	0	0	2
g	91	0.26	0.68	0	0	0	0	3
ptc	69	0.29	0.67	0	0	0	0	2
ti	47	0.64	0.87	0	0	0	1	3
i-IFTA	46	1.17	1.10	0	0	1	2	3
C4d	75	0.48	0.92	0	0	0	1	3
cg	90	0.50	1.03	0	0	0	0	3
mm	91	0.58	0.91	0	0	0	1	3
ah	87	0.77	1.09	0	0	0	2	3
aah	63	0.59	1.09	0	0	0	1	3
cv	89	0.38	0.76	0	0	0	1	3
ci	91	0.62	0.77	0	0	0	1	3
ct	89	0.85	0.68	0	0	1	1	3
Age at Serum Draw	91	47.90	14.39	7	39	50	59.50	72
Age at Biopsy	91	50.31	14.33	7	43	51	61	72
**Biopsy Aggregation**	***n***	**Mean**	**std**	**Min**	**25%**	**50%**	**75%**	**Max**
All Biopsies	112	1.00	0.00	1	1	1	1	1
CC	112	0.39	0.69	0	0	0	1	3
Cav-1 (pg/mL)	112	1099.64	1215.39	3.57	120.17	713.38	1625.97	5882.83
i	112	0.44	0.76	0	0	0	1	3
t	112	0.52	0.84	0	0	0	1	3
v	111	0.15	0.43	0	0	0	0	2
g	112	0.26	0.67	0	0	0	0	3
ptc	87	0.25	0.63	0	0	0	0	2
ti	51	0.63	0.85	0	0	0	1	3
i-IFTA	48	1.17	1.10	0	0	1	2	3
C4d	88	0.45	0.91	0	0	0	0.25	3
cg	111	0.55	1.04	0	0	0	0.5	3
mm	111	0.64	0.95	0	0	0	1	3
ah	108	0.83	1.11	0	0	0	2	3
aah	74	0.69	1.17	0	0	0	1	3
cv	110	0.40	0.78	0	0	0	1	3
ci	112	0.71	0.80	0	0	1	1	3
ct	110	0.91	0.71	0	0	1	1	3
Age at Serum Draw	112	46.54	15.39	7	37.75	48	59	72
Age at Biopsy	112	49.44	15.07	7	40	51	61	75

## Data Availability

Not applicable.

## Supplementary Materials

The following are available online, Figure S1: Decision tree analysis for acute T-cell mediated rejection (ATCMR IA,B); Table S1: Descriptive statistics of acute, chronic and combined rejections; Table S2: Spearman rho correlation coefficients, *p*-values and numbers.

## Sample Availability

Not applicable, biological samples were used in accordance with ethical approval.

## References

[1] Valenzuela N.M., McNamara J.T., Reed E.F. (2014). Antibody-mediated graft injury: Complement-dependent and complement-independent mechanisms. Curr. Opin. Organ Transplant..

[2] Lin C.M., Gill R.G. (2016). Direct and indirect allograft recognition: Pathways dictating graft rejection mechanisms. Curr. Opin. Organ Transplant..

[3] Mikhalski D., Wissing K.M., Ghisdal L., Broeders N., Touly M., Hoang A.D., Loi P., Mboti F., Donckier V., Vereerstraeten P. (2008). Cold ischemia is a major determinant of acute rejection and renal graft survival in the modern era of immunosuppression. Transplantation.

[4] Eltzschig H.K., Eckle T. (2011). Ischemia and reperfusion-from mechanism to translation. Nat. Med..

[5] Fuquay R., Renner B., Kulik L., McCullough J.W., Amura C., Strassheim D., Pelanda R., Torres R., Thurman J.M. (2013). Renal ischemia-reperfusion injury amplifies the humoral immune response. J. Am. Soc. Nephroli..

[6] Rao J., Lu L., Zhai Y. (2014). T cells in organ ischemia reperfusion injury. Curr. Opin. Organ Transplant..

[7] Postalcioglu M., Kaze A.D., Byun B.C., Siedlecki A., Tullius S.G., Milford E.L., Paik J.M., Abdi R. (2018). Association of cold ischemia time with acute renal transplant rejection. Transplantation.

[8] Wu H., Chadban S.J. (2014). Roles of Toll-like receptors in transplantation. Curr. Opin. Organ Transplant..

[9] Sowa G. (2012). Caveolae, caveolins, cavins, and endothelial cell function: New insights. Front. Physiol..

[10] Kang J.W., Lee S.M. (2014). Impaired expression of caveolin-1 contributes to hepatic ischemia and reperfusion injury. Biochem. Biophys. Res. Commun..

[11] Liu M., Wu Y., Liu Y., Chen Z., He S., Zhang H., Wu L., Tu F., Zhao Y., Liu C. (2018). Basic fibroblast growth factor protects astrocytes against ischemia/reperfusion injury by upregulating the caveolin-1/VEGF signaling pathway. J. Mol. Neurosci..

[12] Wang S., Jia L., Zhou H., Wang X., Zhang J. (2008). Caveolin-1 promotes the transformation and anti-apoptotic ability of mouse hepatoma cells. IUBMB Life.

[13] Chen Y.H., Lin W.W., Liu C.S., Hsu L.S., Lin Y.M., Su S.L. (2016). Caveolin-1 expression ameliorates nephrotic damage in a rabbit model of cholesterol-induced hypercholesterolemia. PLoS ONE.

[14] Percy C.J., Pat B.K., Healy H., Johnson D.W., Gobe G.C. (2008). Phosphorylation of caveolin-1 is anti-apoptotic and promotes cell attachment during oxidative stress of kidney cells. Pathology.

[15] Crewe C., Joffin N., Rutkowski J.M., Kim M., Zhang F., Towler D.A., Gordillo R., Scherer P.E. (2018). An endothelial-to-adipocyte extracellular vesicle axis governed by metabolic state. Cell.

[16] Loupy A., Haas M., Solez K., Racusen L., Glotz D., Seron D., Nankivell B.J., Colvin R.B., Afrouzian M., Akalin E. (2017). The Banff 2015 kidney meeting report: Current challenges in rejection classification and prospects for adopting molecular pathology. Am. J. Transplant..

[17] Loupy A., Haas M., Roufosse C., Naesens M., Adam B., Afrouzian M., Akalin E., Alachkar N., Bagnasco S., Becker J.U. (2020). The Banff 2019 kidney meeting report (I): Updates on and clarification of criteria for T cell- and antibody-mediated rejection. Am. J. Transplant..

[18] Tahir S.A., Ren C., Timme T.L., Gdor Y., Hoogeveen R., Morrisett J.D., Frolov A., Ayala G., Wheeler T.M., Thompson T. (2003). Development of an immunoassay for serum caveolin-1: A novel biomarker for prostate cancer. Clin. Cancer Res..

[19] Goes N., Hobart M., Ramassar V., Urmson J., Halloran P.F. (1997). Many forms of renal injury induce a stereotyped response with increased expression of MHC, IFN-gamma, and adhesion molecules. Transplant. Proc..

[20] Daemen M.A.R.C., Veer C.V., Wolfs T.G.A.M., Buurman W.A. (1999). Ischemia/reperfusion-induced IFN-g up-regulation: Involvement of IL-12 and IL-18. J. Immunol..

[21] Zhang X., Beduhn M., Zheng X., Lian D., Chen D., Li R., Siu L.K.S., Marleau A., French P.W., Ichim T.E. (2012). Induction of alloimmune tolerance in heart transplantation through gene silencing of TLR adaptors. Am. J. Transplant..

[22] Cardinal H., Dieude M., Hebert M.J. (2017). The emerging importance of non-HLA autoantibodies in kidney transplant complications. J. Am. Soc. Nephrol..

[23] Zhang Q., Reed E.F. (2016). The importance of non-HLA antibodies in transplantation. Nat. Rev. Nephrol..

[24] Powell J.T., Tsapepas D.S., Martin S.T., Hardy M.A., Ratner L.E. (2013). Managing renal transplant ischemia reperfusion injury: Novel therapies in the pipeline. Clin. Transplant..

[25] Razani B., Zhang X.L., Bitzer M., von Gersdorff G., Bottinger E.P., Lisanti M.P. (2001). Caveolin-1 regulates transforming growth factor (TGF)-beta/SMAD signaling through an interaction with the TGF-beta type I receptor. J. Biol. Chem..

[26] Dai C., Yang J., Liu Y. (2003). Transforming growth factor-beta1 potentiates renal tubular epithelial cell death by a mechanism independent of smad signaling. J. Biol. Chem..

[27] Granata S., Benedetti C., Gambaro G., Zaza G. (2020). Kidney allograft fibrosis: What we learned from latest translational research studies. J. Nephrol..

[28] Young L.H., Ikeda Y., Lefer A.M. (2001). Caveolin-1 peptide exerts cardioprotective effects in myocardial ischemia-reperfusion via nitric oxide mechanism. Am. J. Physiol. Heart Circ. Physiol..

[29] Feng H., Guo L., Song Z., Gao H., Wang D., Fu W., Han J., Li Z., Huang B., Li X.A. (2010). Caveolin-1 protects against sepsis by modulating inflammatory response, alleviating bacterial burden, and suppressing thymocyte apoptosis. J. Biol. Chem..

[30] Sellers S.L., Trane A.E., Bernatchez P.N. (2012). Caveolin as a potential drug target for cardiovascular protection. Front. Physiol..

[31] Yang Y., Ma Z., Hu W., Wang D., Jiang S., Fan C., Di S., Liu D., Sun Y., Yi W. (2016). Caveolin-1/-3: Therapeutic targets for myocardial ischemia/reperfusion injury. Basic Res. Cardiol..

[32] Wang S., Head B.P. (2019). Caveolin-1 in stroke neuropathology and neuroprotection: A novel molecular therapeutic target for ischemic-related injury. Curr. Vasc. Pharmacol..

[33] Chinnakkannu P., Reese C., Gaspar J.A., Panneerselvam S., Pleasant-Jenkins D., Mukherjee R., Baicu C., Tourkina E., Hoffman S., Kuppuswamy D. (2018). Suppression of angiotensin II-induced pathological changes in heart and kidney by the caveolin-1 scaffolding domain peptide. PLoS ONE.

[34] Dragun D., Müller D.M., Bräsen J.H., Fritsche L., Nieminen-Kelhä M., Dechend R., Kintscher U., Rudolph B., Hoebeke J., Eckert E. (2005). Angiotensin II Type 1–Receptor Activating Antibodies in Renal-Allograft Rejection. N. Engl. J. Med..

