# Supplementary material for: Low Pre-Transplant Caveolin-1 Serum Concentrations Are Associated with Acute Cellular Tubulointerstitial Rejection in Kidney Transplantation
Source: Molecules. 2021 Apr 30;26(9):2648. doi: 10.3390/molecules26092648 (PMC8125494; doi:10.3390/molecules26092648)
Supplement: Supplementary file 1 [file molecules-26-02648-s001.zip › Suppl_table_1.docx]

| Rejection Categories | n | mean | std | min | 25% | 50% | 75% | max |
| --- | --- | --- | --- | --- | --- | --- | --- | --- |
| ATCMR, 1 Biopsy per Patient | 91 | 1.27 | 1.21 | 0 | 0 | 2 | 2 | 3 |
| ATCMR IA,B; 1 Biopsy per Patient | 91 | 1.10 | 1.13 | 0 | 0 | 2 | 2 | 3 |
| ATCMR IIA,B,III; 1 Biopsy per Patient | 91 | 0.13 | 0.34 | 0 | 0 | 0 | 0 | 1 |
| ATCMR, all Biopsies | 111 | 1.14 | 1.22 | 0 | 0 | 0 | 2 | 3 |
| ATCMR IA,B; all Biopsies | 112 | 0.96 | 1.11 | 0 | 0 | 0 | 2 | 3 |
| ATCMR IIA,B,III; all Biopsies | 111 | 0.13 | 0.33 | 0 | 0 | 0 | 0 | 1 |
| AABMR, 1 Biopsy per Patient | 62 | 0.13 | 0.34 | 0 | 0 | 0 | 0 | 1 |
| CAABMR, 1 Biopsy per Patient | 62 | 0.10 | 0.30 | 0 | 0 | 0 | 0 | 1 |
| CIABMR, 1 Biopsy per Patient | 62 | 0.18 | 0.39 | 0 | 0 | 0 | 0 | 1 |
| AABMR, all Biopsies | 74 | 0.14 | 0.34 | 0 | 0 | 0 | 0 | 1 |
| CAABMR, all Biopsies | 74 | 0.11 | 0.31 | 0 | 0 | 0 | 0 | 1 |
| CIABMR, all Biopsies | 74 | 0.19 | 0.39 | 0 | 0 | 0 | 0 | 1 |
| CATCMR, 1 Biopsy per Patient | 46 | 0.26 | 0.44 | 0 | 0 | 0 | 0.75 | 1 |
| CATCMR, all Biopsies | 48 | 0.25 | 0.44 | 0 | 0 | 0 | 0.25 | 1 |

Supplemental table 1: Acute, chronic and combined rejections. Abbreviations according to Figure 1. Data is displayed on the patients’ level and biopsy-based.
