# Supplementary material for: Low Pre-Transplant Caveolin-1 Serum Concentrations Are Associated with Acute Cellular Tubulointerstitial Rejection in Kidney Transplantation
Source: Molecules. 2021 Apr 30;26(9):2648. doi: 10.3390/molecules26092648 (PMC8125494; doi:10.3390/molecules26092648)
Supplement: Supplementary file 1 [file molecules-26-02648-s001.zip › Suppl_table_1.pdf]

Rejection Categories	n	mean	std	min	25%	50%	75%	max
ATCMR, 1 Biopsy per Patient	91	1.27	1.21	0	0	2	2	3
ATCMR IA,B; 1 Biopsy per Patient	91	1.10	1.13	0	0	2	2	3
ATCMR IIA,B,III; 1 Biopsy per Patient	91	0.13	0.34	0	0	0	0	1
ATCMR, all Biopsies	111	1.14	1.22	0	0	0	2	3
ATCMR IA,B; all Biopsies	112	0.96	1.11	0	0	0	2	3
ATCMR IIA,B,III; all Biopsies	111	0.13	0.33	0	0	0	0	1
AABMR, 1 Biopsy per Patient	62	0.13	0.34	0	0	0	0	1
CAABMR, 1 Biopsy per Patient	62	0.10	0.30	0	0	0	0	1
CIABMR, 1 Biopsy per Patient	62	0.18	0.39	0	0	0	0	1
AABMR, all Biopsies	74	0.14	0.34	0	0	0	0	1
CAABMR, all Biopsies	74	0.11	0.31	0	0	0	0	1
CIABMR, all Biopsies	74	0.19	0.39	0	0	0	0	1
CATCMR, 1 Biopsy per Patient	46	0.26	0.44	0	0	0	0.75	1
CATCMR, all Biopsies	48	0.25	0.44	0	0	0	0.25	1

Supplemental table 1: Acute, chronic and combined rejections. Abbreviations according to Figure 1. Data is displayed on the patients' level and biopsy-based.
