# Supplementary figures and images for: Low Pre-Transplant Caveolin-1 Serum Concentrations Are Associated with Acute Cellular Tubulointerstitial Rejection in Kidney Transplantation
Source: Molecules. 2021 Apr 30;26(9):2648. doi: 10.3390/molecules26092648 (PMC8125494; doi:10.3390/molecules26092648)

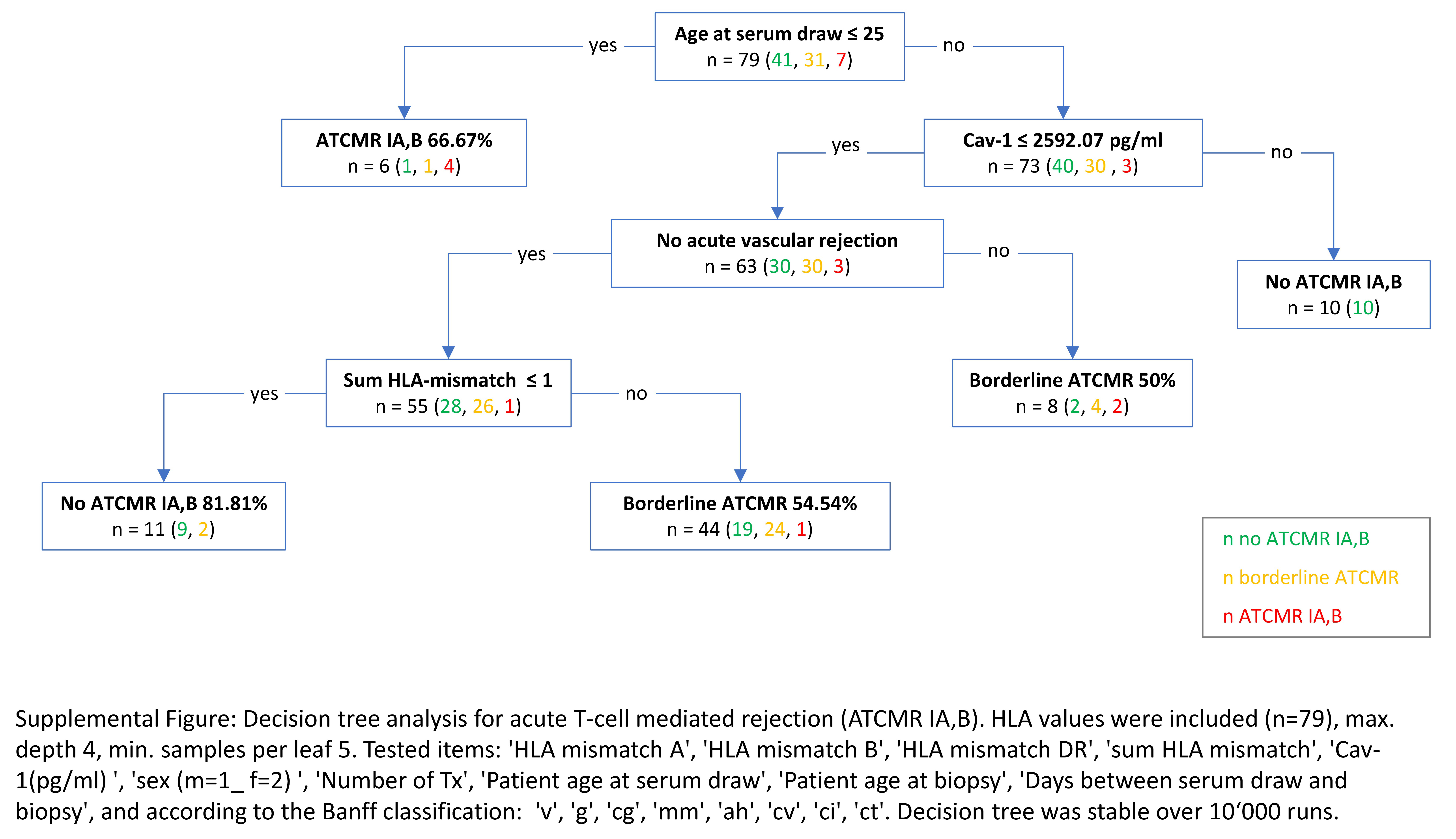

Supplement: Supplementary file 1 [file molecules-26-02648-s001.zip › Suppl_Figure_incl_description.jpg]
